# Traumatic Injury Causing Intraperitoneal Hemorrhage of an Occult Pheochromocytoma

**DOI:** 10.1155/2012/342819

**Published:** 2012-10-14

**Authors:** Arpit Amin, Saptarshi Biswas, Francis Baccay

**Affiliations:** Department of Surgery, New York Medical College, Westchester Medical Center, Valhalla, NY 10595, USA

## Abstract

Pheochromocytoma is a rare catecholamine-secreting tumor derived from chromaffin cells. The diagnosis is usually suggested by classic history in a symptomatic patient, presence of a strong family history in a patient, or discovery of an incidental mass on imaging in an asymptomatic patient. Traumatic hemorrhage into an occult pheochromocytoma presenting as hypovolemic shock is a rare presentation of pheochromocytoma. We report a case of a 48-year-old female, who presented in hypovolemic shock due to unilateral adrenal hemorrhage secondary to a fall from horse. Computed tomographic imaging revealed that the source of the hypovolemic shock was hemorrhagic right adrenal mass with active extravasation. The patient underwent emergent selective arterial embolization of right superior adrenal artery and a small adrenal branch from the right renal artery to control the hemorrhage. The patient subsequently progressed to sepsis and MODS, needing multiple surgical procedures and a protracted recovery in the ICU. In the ICU, the patient suffered from rapid cyclic fluctuation of her systolic blood pressure and was subsequently diagnosed with pheochromocytoma secondary to traumatic hemorrhage. We discuss this rare case along with the presentation and diagnostic workup of this critically ill patient with a previously undiagnosed pheochromocytoma.

## 1. Introduction

Pheochromocytoma is a rare catecholamine-secreting tumor derived from chromaffin cells. The diagnosis is usually suggested by classic symptoms of headaches, paroxysmal or essential hypertension, sweating, and tachycardia in a symptomatic patient. We present an unusual case of a 48-year-old female with blunt trauma presenting with intraperitoneal hemorrhage due to actively bleeding unilateral right-sided adrenal mass. 

## 2. Case Presentation

A 48-year-old female sustained a fall while riding her horse. The patient was evaluated at a community level 2 trauma center. The patient was transferred to our institution once the initial trauma evaluation revealed perihepatic hematoma, left internal carotid artery dissection, and bilateral pulmonary contusions. En route, the patient became hemodynamically unstable requiring crystalloids and packed red blood cell transfusion.

**Figure 1:**
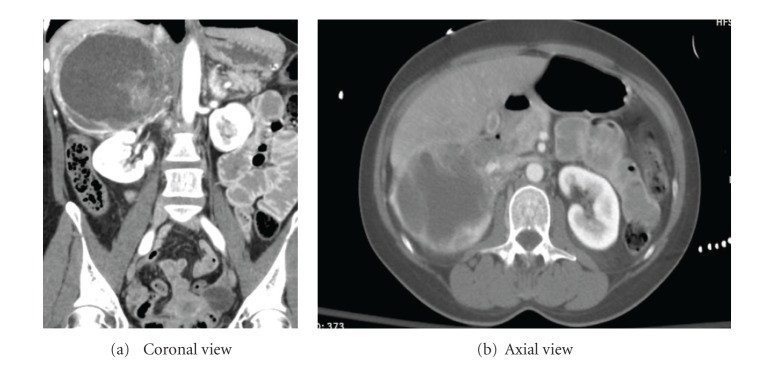
Two views of 11.4 cm∗1.4 cm∗1.7 cm adrenal mass actively extravasating right adrenal mass.

**Figure 2:**
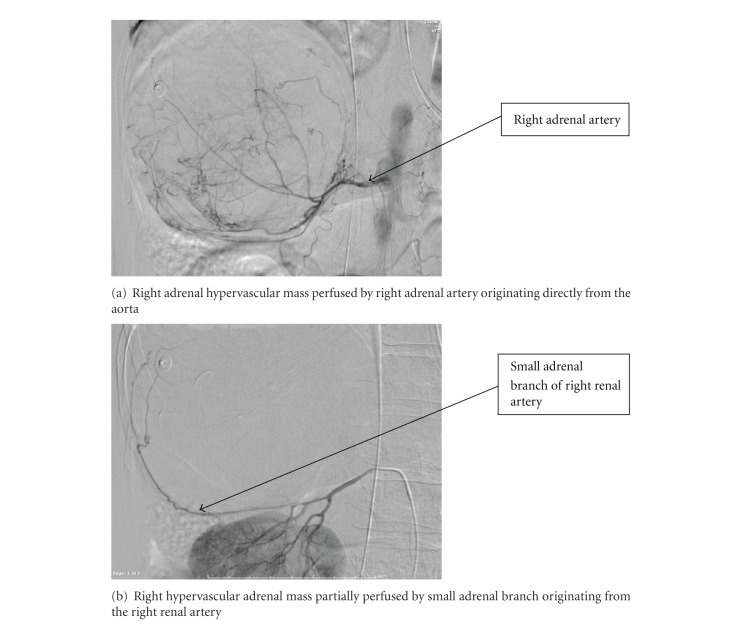
Angiogram of right adrenal mass.

Vital signs on arrival were T 95.5, HR 140, BP 107/65, and O2 sat 99% on assist-controlled ventilation. Repeat imaging at our institution revealed a heterogeneously enhancing mass with active extravasation suggesting hemorrhagic adrenal mass rather than inferior hepatic lobe hematoma (Figures 1(a) and 1(b)). The patient underwent emergent angiography with selective arterial embolization of right superior adrenal artery and a small adrenal branch from the right renal artery to control the hemorrhage (Figure 2). 

The patient was admitted to trauma intensive care unit for further resuscitation and stabilization. Profound hypovolemic shock led to myocardial infarction and acute kidney injury prior to control of her hemorrhage. Peritoneal signs were revealed on clinical examination on hospital day 2 leading to emergent exploratory laparotomy. Extended right hemicolectomy was performed for ischemic colitis and the abdomen was temporarily left open. Subsequently, on reexploration, an ileocolic side-to-side anastomosis was performed and abdominal wall closure was performed using component separation. On hospital day 24, patient developed an episode of melena resulting in another episode of hypovolemic shock requiring vasopressors. Nasogastric tube lavage ruled out any upper GI bleeding. Patient underwent sigmoidoscopy and exploratory laparotomy. These procedures did not reveal any source of lower GI bleeding or any evidence of anastomotic bleeding. The patient's blood pressure became stable on hospital day 28 with no further vasopressor requirement. 

On hospital day 30, the patient developed rapid cyclic fluctuations in systolic blood pressure from 50 to 240. The patient denied any symptoms while these fluctuations in blood pressure were noted. The cyclic fluctuation was finally controlled after an interval of 15 minutes with the use of labetalol. 24-hour urine catecholamines were sent and found to be as follows: normetanephrine 3195 *μ*g (normal range: 88–649), metanephrine 5238 *μ*g (normal range: 182–739), norepinephrine 27 *μ*g (normal range: 15–100), epinephrine 16 *μ*g (normal range: 9–21), and vanillylmandellic acid 8.4 *μ*g (normal range: <6.0). Diagnosis of functional pheochromocytoma was made. 

A detailed history was performed to elucidate prior history of hypertension and any symptoms related to pheochromocytoma. The patient did not have any past history of hypertension or cancer in her family. Detailed physical examination did not reveal any evidence of atypical cutaneous findings like café-au-lait spots or axillary freckling. Further laboratory evaluation showed low corrected calcium (8.3 mg/dL, normal range 8.6–10.2 mg/dL), low phosphate (1.8 mg/dL, normal range 2.3–4.7 mg/dL), high PTH 67.9 pg/mL (normal range 7–53.0 pg/mL), low Vitamin D (25-OH) 19 ng/mL (normal range 30–80 ng/mL), and normal Vitamin D (1,25-OH_2_) 22 pg/mL (normal range 18–72 pg/mL). The patient underwent a sestamibi scan, which revealed no parathyroid pathology. Thyroid ultrasound revealed 0.3 cm∗0.3 cm∗0.3 cm hypoechoic nodule in the right lobe and 0.5 cm∗0.4 cm∗0.4 cm hypoechoic nodule in the left lobe. CEA and calcitonin levels were within normal limits. Having ruled out multiple endocrine neoplasia syndrome in this patient, we proceeded to control the patient's symptoms with a nonselective *α*-blocker. Thereafter, the patient was put on a long-acting *β*-blocker for planned resection of pheochromocytoma. 

**Figure 3:**
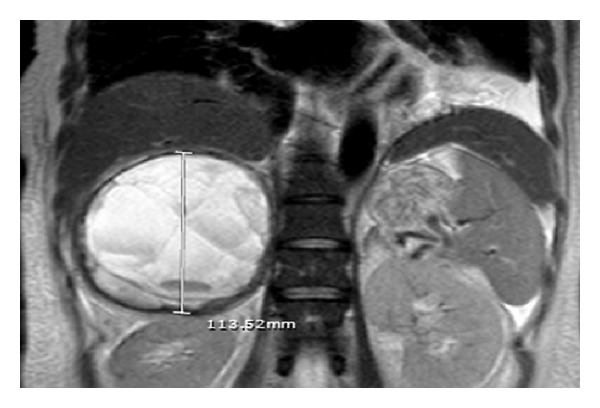
MRI abdomen (T2-weighted) of right adrenal mass.

MRI of the abdomen was obtained for preoperative planning and showed 10.3 cm∗9.9 cm∗11.4 cm right adrenal mass with solid components anteriorly and cystic components posteriorly (Figure 3). There was no evidence of inferior vena cava involvement.

Exploratory laparotomy was performed and the right adrenal mass was located after extensive lysis of adhesions. The right triangular ligament and the right posterior segment of the liver were involved (segment 7). As a result, intraoperative decision was made to perform right adrenalectomy along with segment 7 hepatic resection. The mass was sent for pathology. The patient tolerated the procedure well and was discharged home on fifth post-operative day. 

**Figure 4:**
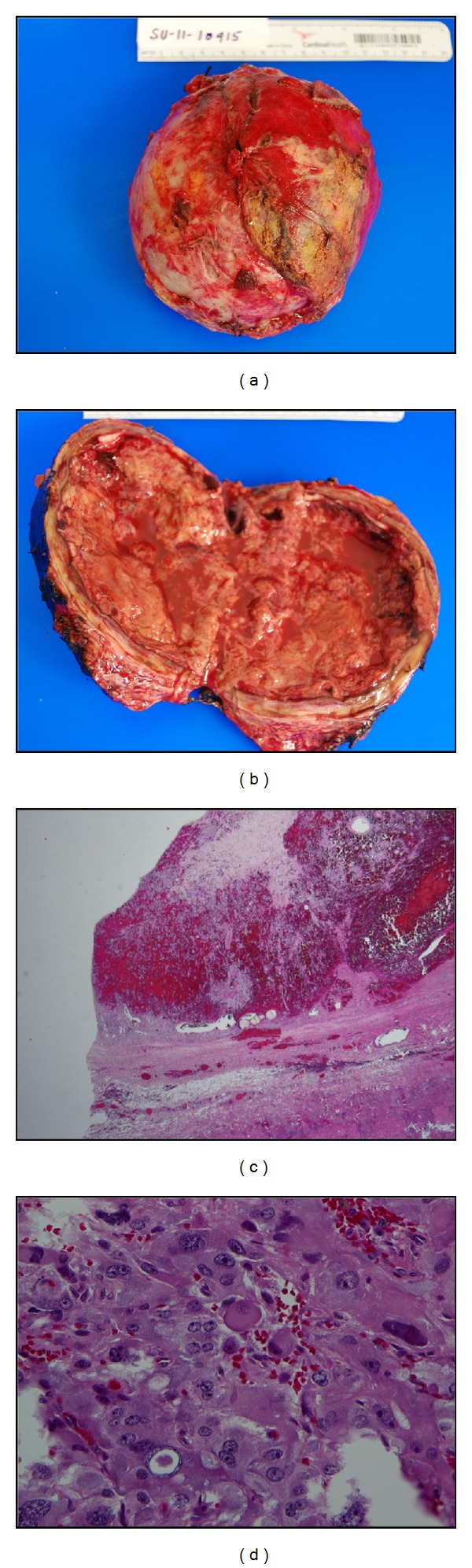
(a) and (b) Enlarged cystic adrenal mass filled with tan red necrotic material. (c) Hemorrhagic and fibrotic components within adrenal medulla mass (H & E; 100X). (d) Tumor cells are large with abundant pink to mauve cytoplasm and arranged in nests with capillaries in between (H & E; 400X).

**Figure 5:**
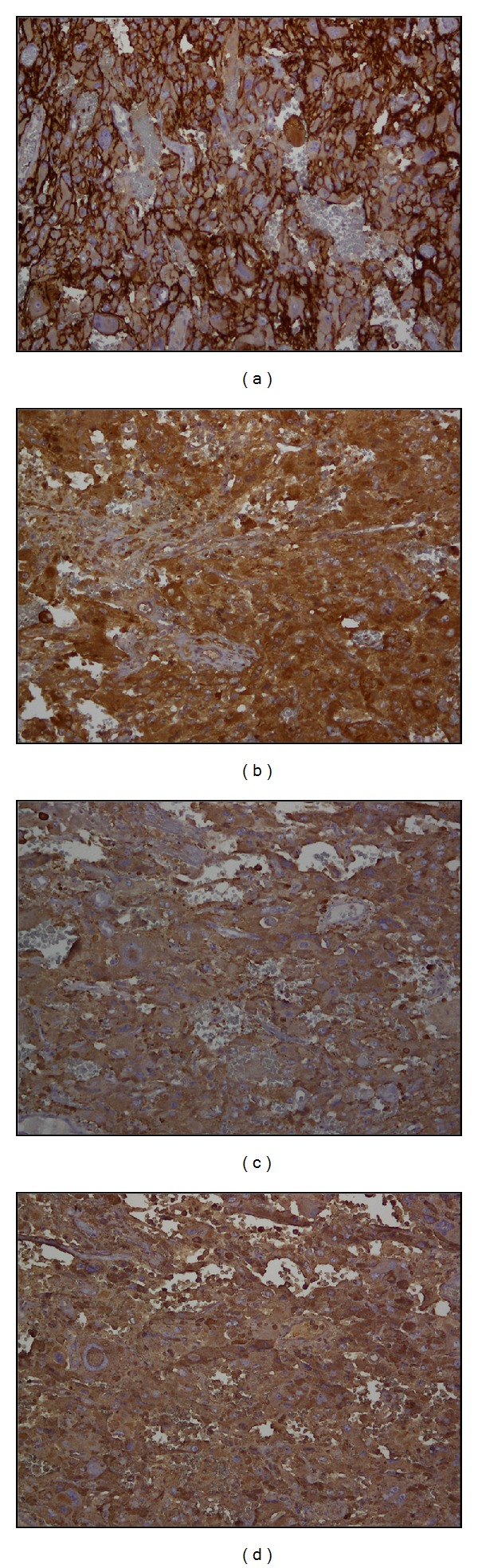
Membranous staining for CD56 (a) and cytoplasmic staining for neuron-specific enolase (b), synaptophysin (c), and chromogranin (d) in tumor cells (H & E; 200X).

Pathology revealed 13.0 cm∗12.5 cm∗4.5 cm size right adrenal mass consistent with a diagnosis of pheochromocytoma. Approximately, 90% of the tumor showed necrosis with hemorrhage, fibrosis, and hyalinization with a peripheral viable rim of the tumor (Figure 4). No vascular invasion was found. Focal rim of benign hepatocytes was identified. Immunohistochemically, the tumor stained positive for CD56, chromogranin, synaptophysin, and neuron-specific enolase (Figure 5). 

## 3. Discussion

Our patient sustained a traumatic fall leading to adrenal gland hemorrhage causing hypovolemic shock and subsequently manifested symptoms of pheochromocytoma once the patient's hemorrhage was controlled with embolization. There have been multiple case reports in the literature describing hemorrhagic shock due to spontaneous rupture of adrenal pheochromocytoma [1]. However, the mechanism of blunt trauma causing hemorrhagic shock and subsequent, delayed activation of a “latent” pheochromocytoma once the hemorrhagic shock is controlled is a unique and rare presentation of pheochromocytoma. 

The prevalence of pheochromocytoma is about 0.2% based on autopsy studies and the mean age at diagnosis of pheochromocytoma is about 40 years [2]. Pheochromocytoma is traditionally described by the “rule of tens”—10% are bilateral, 10% are extra-adrenal, 10% are hereditary, and 10% are malignant [3]. Pheochromocytomas are catecholamine-secreting, well-vascularized tumors that arise from chromaffin cells derived from the neural crest cells. 

The clinical presentation of pheochromocytoma is highly variable. The classic triad of symptoms consisting of episodic palpitations, headaches, and profuse sweating in the presence of hypertension, either persistent or paroxysmal, usually raises the suspicion of pheochromocytoma [2]. This classic presentation occurs in only 85%–90% of cases and the diagnosis is fairly straightforward [4]. However, the atypical presentation of pheochromocytoma makes it challenging from the diagnostic and therapeutic point of view. For instance, there are cases reported in the literature of atypical presentations of pheochromocytoma in the form of cardiogenic shock due to dilated cardiomyopathy and hemorrhagic shock due to spontaneous intraperitoneal hemorrhage [1, 3, 5].

Once clinical presentation raises suspicion for pheochromocytoma, the diagnosis can be made with help of biochemical assays that determine levels of catecholamines and metanephrines in the plasma and the urine. Plasma metanephrines have been found to have the highest sensitivity while 24-hour urine vanillylmandellic acid has been found to have the highest specificity for the diagnosis of pheochromocytoma [6]. It should be noted that the false positives for these biochemical test may occur in the presence of acute traumatic injury or in presence of critical illness. The patient in our case was critically ill when the clinical manifestations of her “latent” pheochromocytoma occurred. In order to rule out false positives, sequential 24-hour urine catecholamine levels and plasma catecholamine levels were obtained once the patient was more stable and were found to be persistently elevated. 

Following biochemical confirmation, CT scan with iodinated contrast or MRI (T2-weighted) imaging with gadolinium contrast can be utilized to determine the location and extent of pheochromocytoma [2]. In our case, CT scan with IV contrast was performed in the acute trauma setting and the finding of actively extravasating hemorrhagic component of adrenal mass was noted. Once the diagnosis of pheochromocytoma was established, MRI scan (T2-weighted) was performed for preoperative planning and demonstrated cystic as well as solid changes. The findings of necrosis on pathology and hemorrhagic as well as cystic components on imaging are not uncommon for pheochromocytoma [4].

About 25% of patients with pheochromocytoma have an associated hereditary syndrome. As a result, once a diagnosis of pheochromocytoma is made, it is important to rule out any evidence of hereditary syndromes like multiple endocrine neoplasia type 2, neurofibromatosis type 1, and von Hippel-Landau syndrome in the patient. Our patient did not have any clinical manifestations associated with hereditary syndromes and laboratory testing was performed to rule out presence of these hereditary syndromes before offering definitive surgical management of pheochromocytoma. 

Treatment of a patient diagnosed with pheochromocytoma involves medical optimization with alpha-adrenergic blockade and hydration followed by adequate beta-blockade. Once medical optimization is satisfactory, transperitoneal or retroperitoneal approach can be utilized to complete surgical removal of pheochromocytoma. 

**Figure 6:**
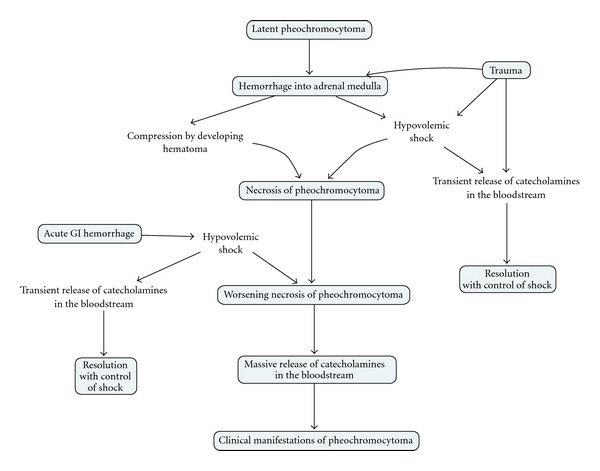
Pathophysiology of activation of “latent” pheochromocytoma.

The pathophysiologic mechanism of activation of a “latent” pheochromocytoma in the setting of trauma in our patient can be postulated as shown in Figure 6. 

In reviewing the literature we came across one reported case of latent pheochromocytoma diagnosed in the setting of blunt trauma. In the case reported by May and colleagues, the patient presented with traumatic hemorrhagic shock requiring embolization followed immediately by massive catecholamine surge resulting in persistent hypertension, which was found to be refractory to treatment with nitroprusside and esmolol and subsequently led the authors to pursue emergent adrenelectomy to help alleviate the patient's symptoms [7]. This emergent approach was successful in their case. 

Our management of latent pheochromocytoma diagnosed in the setting of acute traumatic hemorrhagic shock was different. In the acute trauma setting, it should be noted that hemodynamically unstable patient with actively hemorrhaging adrenal mass will most likely undergo embolization at most trauma centers with interventional radiology capability. Once the acute hemorrhage has been controlled, latent pheochromocytoma has a potential to become activated. In our case, this occurred after the patient suffered another episode of hypovolemic shock due to gastrointestinal bleeding. In the case reported by May and colleagues, it occurred immediately after the source of hemorrhage was controlled. Regardless of the presentation, we recommend controlling the hypertensive paroxysm in the acute trauma setting with intravenous labetalol. The review of the literature regarding the management of spontaneous hemorrhagic pheochromocytoma reveals that emergent surgical management is associated with a higher mortality rate compared to elective surgical management [5, 8]. Based on this data, we favored the sequential approach of medically optimizing the patient with alpha-blockade followed by beta-blockade and thereafter proceeding with an elective open transperitoneal unilateral adrenelectomy. The open approach was favored over the laparoscopic approach in this case due to patient's recent history of exploratory laparotomy and proximity of the mass to the liver on pre-operative MRI.
